# Trehalose/sodium hyaluronate eye drops in post-cataract ocular surface disorders

**DOI:** 10.1007/s10792-021-01869-z

**Published:** 2021-05-06

**Authors:** Carlo Cagini, Giovanni Torroni, Marco Mariniello, Giampiero Di Lascio, Gianluca Martone, Angelo Balestrazzi

**Affiliations:** 1 Department of Medicine and Surgery, Ophthalmology Section, S. Maria Della Misericordia Hospital, University of Perugia, Perugia, Italy; 2 Ophthalmology Unit, Misericordia Hospital, USL Toscana Sud Est, Grosseto, Italy

**Keywords:** Cataract surgery, Dry eye, Trehalose/sodium hyaluronate/carbomer eye drops, Post-operative

## Abstract

**Purpose:**

Prospective, randomised, open-label, comparative study to evaluate efficacy of trehalose/sodium hyaluronate eye drops for post-operative discomfort and tear film stability in patients undergoing cataract surgery.

**Methods:**

Patients with healthy ocular surface, subclinical, or mild dry eye were enrolled. Tear breakup time (TBUT), Schirmer test, dry eye symptoms, corneal fluorescein staining (CFS), and ocular surface disease (OSDI) evaluation were performed pre-operatively and at two and four weeks after surgery. Patients were assigned to receive trehalose/sodium hyaluronate eye drops b.i.d (Group A), or 0.9% unpreserved sodium chloride eye drops b.i.d for 4 weeks (Group B).

**Results:**

One hundred and thirty-five patients were randomised, 66 patients in Group A (73.2 ± 4.5 years) and 69 patients in Group B (74.3 ± 3.8 years), 60.8% females. Fifteen patients (8 Group A) were lost at follow-up. Pre-operatively, no between-group differences were observed, and TBUT increased in Group A between the pre-operative and 2 and 4 week evaluations and was higher in group A than in Group B at 4 weeks. Schirmer test and CFS showed an improvement only in Group A four weeks post-operatively. In Group A an improvement was observed after two and four weeks in foreign body and puncture sensation, whilst a difference in blinking discomfort was observed after four weeks. In Group B we observed an improvement in puncture sensation two and four weeks after surgery. Mean OSDI scores differences between the two groups were significant at four weeks.

**Conclusions:**

Trehalose/sodium hyaluronate eye drops were effective in reducing signs and symptoms of dry eye and improving tear film stability

## Introduction

Cataract surgeons are primarily focused on ensuring high-quality surgery and optimal post-operative visual acuity but from a patient’s point of view, intra and post-operative ocular discomfort may be even more important. After cataract surgery, a large number of patients report dry eye symptoms, including conjunctival hyperaemia, ocular surface irritation and itching, tearing, and foreign body sensation [1–3]. Discomfort can last for several weeks after surgery and can reduce the quality of life of patients by affecting their everyday activities and lead to poor patient satisfaction [4]. This is particularly true in elderly patients where the prevalence of pre-operative subclinical or clinical dry eye is high and cataract surgery aggravates these signs and symptoms. After cataract surgery, various factors may contribute to tear film instability, including the toxic effect of topical anaesthesia, the operating microscope light, and post-operative medications containing preservatives [2, 3, 5]. Consequently, post-cataract dry eye management is becoming increasingly important, [3, 5, 6] with the recommended treatment being non-preserved artificial tears following phacoemulsification. In many cases the use of artificial tears improves the satisfaction of patients in reducing post-operative discomfort [2].

Thealoz*®* gel are eye drops composed of a natural disaccharide (trehalose) and a natural biopolymer (sodium hyaluronate [HA]). Whilst trehalose is recognised for its bio and osmo-protective activity, HA has been shown to be useful in dry eye patients for its ability to retain water and hydrate the ocular surface, which increases the tear film thickness and improves the ocular surface index. [7, 8] This trehalose and HA combination is of particular interest due to its protection of the ocular surface [9, 10]. The aim of this study was to evaluate the efficacy of trehalose 3%/HA 0.15% eye drops as a gel compared to unpreserved 0.9% sodium chloride eye drops in restoring tear film stability and relieving ocular discomfort after cataract surgery.

## Methods

### Study design and patients

This prospective, randomised, open-label, comparative study involved adult patients undergoing uneventful unilateral cataract surgery and intra-ocular lens implantation recruited at the Ophthalmology Section of the University of Perugia’s Department of Biomedical and Surgical Sciences and at the Ophthalmology Department of Grosseto Hospital. The study was approved by local Ethics Committees (CEAS Umbria, Prot. n. 3411/19) and conducted in accordance with Good Clinical Practice and the ethical principles laid down in the 1964 Declaration of Helsinki and its amendments. Written informed consent was obtained from each patient.

### Study population

Patients of both sexes aged > 18 years undergoing unilateral cataract surgery and intra-ocular lens implantation, with a healthy ocular surface, subclinical or mild dry eye disease (DED) and who used artificial tears only occasionally were eligible for inclusion. The main exclusion criteria were previous ocular surgery; a history of trauma or ocular inflammation, moderate or severe DED, or any corneal pathologies; anatomical or functional eyelid abnormalities; ocular hypertension or glaucoma; the concurrent administration of topical eye drops other than those included in the study protocol and/or treatment with eye drops for DED within 1 week before the screening visit and before surgery; any systemic drug that may contribute to DED such as anti-histamines, decongestants, antidepressants, and spasmolytics; and the use of contact lenses. The administration of systemic corticosteroids or non-steroidal anti-inflammatory drugs was not allowed during the study. Patients were withdrawn from the study if they experienced any surgery-related complications such as posterior capsule rupture, vitreous loss, or post-operative anterior uveitis.

### Assessments

The following assessments were performed pre-operatively, and at two weeks and four weeks after surgery: Tear Breakup Time (TBUT); Schirmer test; VAS questionnaire including three questions each of which with an answer scale from 0 (no symptom) to 10 (the worst symptom) for foreign body sensation, pricking sensation and discomfort in blinking, respectively; the ocular surface disease index (OSDI); and the corneo-conjunctival epithelial damage after fluorescein staining (CFS) (Oxford score). The corneal staining grade was based on the National Eye Institute staining grid from 0 (normal) to 3 (severe).

### Surgical procedures

On the day of surgery, patients were randomly assigned into Group A (study group) or Group B (control group) in a 1:1 ratio using a computer-generated randomisation list. The examiner who carried out the post-operative assessment and the patient did not know in which group they were assigned. The surgery was performed by one experienced surgeon in Perugia (C.C.) and one in Grosseto (A.B.). The patients’ pupils were dilated by a phenylephrine and tropicamide insert (Mydriasert®, Laboratoires Théa, France) about 60 min before surgery. Each procedure was performed under topical anaesthesia (lidocaine 4%) and standard phacoemulsification was carried out through a temporal clear cornea incision. Once discharged, all patients were treated with ofloxacin eye drops (Oftaquix®, Santen, Italy) *q.i.d* for the first week, chloramphenicol 0,25% and dexamethasone 0.13% eye drops (Betabioptal®, Laboratoires Théa) *q.i.d* for two weeks, and preservative-free 1% diclofenac eye drops (Voltaren Oftabak®, Laboratoires Théa) *q.i.d* for four weeks. Patients in Group A received a fixed combination of unpreserved trehalose and HA eye drops (Thealoz® gel, Laboratoires Théa), whilst patients in Group B received unpreserved 0.9% sodium chloride eye drops (Hydrabak®, Laboratoires Théa) *b.i.d* for four weeks.

### Statistical analysis

The study planned to enrol at least 130 patients, 65 in each treatment arm, which was determined from literature data with an expected dropout rate of about 10%. This sample size would provide 80% power to assess the non-inferiority of the study treatment compared with the control treatment.

The continuous variables were described as mean value ± SD and were analysed using ANOVA for repeated measures, taking pre-treatment values as covariate. The effect of treatment on variables of interest over time was described by mean value ± SD and analysed using multivariate analysis of variance for repeated measures, with time, treatment, and their interaction as effects. Between-group comparisons were performed using independent* t* tests or Mann–Whitney *U* tests. Within group comparisons were made using paired t tests or Wilcoxon signed-rank tests where appropriate after Shapiro–Wilk tests were used to check data normality. A *p*-value < 0.05 was considered statistically significant.

## Results

One hundred and thirty-five patients (135 eyes) were enrolled in this study, and they were randomised 66 patients in Group A and 69 patients in Group B. The mean ± SD age was 73.2 ± 4.5 years in Group A and 74.3 ± 3.8 years in Group B with 60.8% females and 39.2% males. Fifteen patients were lost at follow-up, 8 in Group A and 7 in Group B for intraoperative complications or lack of presentation at follow-up examinations. At the pre-operative visit, all studied parameters were similar between treatment groups, with no statistically significant between-group differences. The post-operative treatment was well tolerated and none of the patients reported any adverse event.

In Group A, there was a statistically significant improvement in TBUT compared to the pre-operative assessment, which increased from 8 + 2.49 to 10.1 + 2.24 at week 2 (*p* = 0.0007) and to 12.2 + 3.15 at week 4 (*p* = 0.0001). However, in Group B, this improvement in TBUT post-operatively was not observed (Week 2; *p* = 0.8870*,* Week 4; *p* = 0.7825). (Table 1). In addition, there was a statistically significant difference between Group A and Group B at Week 4 (*p* = 0.024). Similar improvements were also observed in Group A at Week 4 for the Schirmer test (*p* = 0.0010) and CFS (*p* = 0.002) (Table 2 and Table 3). In Group B, there was no statistically significant difference at any timepoint post-operatively for the Schirmer test or CFS, whilst there was a statistically significant difference between Group A and Group B at Week 4 in CFS (*p* = 0.015).

**Table 1:** Mean TBUT pre-operatively and 2 weeks and 4 weeks post-operatively

	Pre op	Week 2	Week 4
Group A	8 + 2.49^a^	10.1 + 2.24^a,b^	12.2 + 3.15^b,c^
Group B	10 + 3.88	9.85 + 3.71	9.7 + 3.55^c^

^a^*P *< 0.05

^c^*P *< 0.05

^c^*P *< 0.05

**Table 2:** Mean Schirmer test pre-operatively and 2 weeks and 4 weeks post-operatively

	Pre op	Week 2	Week 4
Group A	11.75 + 2.97^a^	12.75 + 2.99	14.6 + 3.38^a^
Group B	13.05 + 4.44	11.55 + 3.8	12.65 + 3.95

^a^*P *< 0.05

**Table 3:** Corneal fluorescein staining scores (Oxford Score) at 2 weeks and 4 weeks

	Pre op	Week 2	Week 4
Group A	2.25 + 0.55^a^	1.95 + 0.51	1.5 + 0.51^ab^
Group B	2.15 + 0.49	1.85 + 0.49	1.95 + 0.57^b^

^a^*P *< 0.05

^b^*P *< 0.05

The mean OSDI scores improved significantly in both groups post-operatively, with a statistically significant difference in both groups at Week 2 and Week 4 (Table 4), whilst a statistically significant difference between Group A and Group B was observed at Week 4 (*p* = *0.001*). In the study group, two and four weeks after surgery we observed an improvement of foreign body sensation and puncture sensation, with a statistically significant difference respect to pre-operative value (FB^d^*p* = 0,0092 and *p* = 0,0018; PS: *p* = 0,0052 and *p* = 0,0092, respectively) (Tables 5 and 6) whilst discomfort in blinking showed an improvement trend and 4 weeks after surgery the difference with the pre-operative value has become statistically significant (*p* = 0,0724 after two weeks and *p* = 0,0421 after four weeks) (Table 7). In control group we observed an improvement only in puncture sensation two and four weeks after surgery.

**Table 4:** Mean OSDI values pre-operatively and 2 weeks and 4 weeks post-operatively

	Pre op	Week 2	Week 4
Group A	8.2 + 7.9^a,b^	2.2 + 3.4^a^	1.2 + 1.9^b,c^
Group B	9.3 + 9.4^d,e^	4.1 + 4.1^d^	4.8 + 3.4^c,e^

^a^*P *< 0.05

^b^*P *< 0.05

^c^*P *< 0.05

^d^*P *< 0.05

^e^*P *< 0.05

**Table 5:** Foreign body sensation pre-operatively and at 2 weeks and 4 weeks

	FBS		
	Pre op	2 week	4 week
Group A	1,72 + 1,73^a,b^	0,62 + 0,9^a^	0,32 + 0,51^b,c^
Group B	0,9 + 1,25	1,3 + 1,33	1,57 + 1,41^c^

*FBS* foreign body sensation

^a^*P *< 0.05

^b^*P *< 0.05

^c^*P *< 0.05

**Table 6:** Puncture sensation pre-operatively and at 2 weeks and 4 weeks

	PS		
	Pre op	2 week	4 week
Group A	1,45 + 1,99^a,b^	0,2 + 0,52^a^	0,25 + 0,55^b,e^
Group B	0,9 + 1,04^c,d^	0,42 + 0,81^c^	0,2 + 0,53^d,e^

*PS* puncture sensation

^a^*P *< 0.05

^b^*P *< 0.05

^c^*P *< 0.05

^d^*P *< 0.05

^e^*P *< 0.05

**Table 7:** Blinking discomfort pre-operatively and at 2 weeks and 4 weeks

	BD		
	Pre op	2 week	4 week
Group A	0,5 + 0,88^a^	0,1 + 0,31	0,1 + 0,31^a,b^
Group B	0,4 + 1,19	0,2 + 0,53	0,55 + 0,99^b^

*BD* blinking discomfort

^a^*P *< 0.05

^b^*P *< 0.05

## Discussion

In this prospective, randomised, open-label, comparative study, an improvement was observed for clinical assessments and subjective ocular symptoms associated with dry eye at Week 2 and Week 4 post-operatively for patients using a fixed combination of unpreserved trehalose and HA eye drops, which were statistically significant at Week 4. There was also a statistically significant difference between treatment groups at Week 4 in favour of trehalose and HA compared to 0.9% sodium chloride for all assessments, except for Schirmer test.

Cataract surgery is an important and effective technique used to improve a patient’s visual acuity [11]. However, a significant number of patients report symptoms associated with dry eye following surgery with 87% of patients describing symptoms in the first post-operative week, 35% after three months and 10% of patients developing chronic dry eye [12]. As these symptoms can affect a patients’ quality of life [13], it is important to manage them appropriately to improve the clinical outcome and patient satisfaction following cataract surgery [14–16].

A variety of treatments are available for post-operative dry eye, and with a deeper understanding of its pathophysiology, the optimal intervention has shifted from simply hydrating the ocular surface with artificial tears to applying eye drops with lubricating and cell-binding properties. In our study, the use of combined 3% trehalose/0.15% sodium hyaluronate eye drops led to a marked improvement in the signs and symptoms of dry eye. In particular, a significant improvement was observed in the TBUT, which is considered an index of tear film instability, both post-operatively and compared to 0.9% sodium chloride eye drops. This greater stability of the tear film may be a consequence of lower evaporation and reduced tear film hyperosmolarity, which when increased are evidence of dry eye [17]. The improved stability of the tear film in Group A was also accompanied by a reduction in CFS, an indicator of corneal tissue cell damage, and by a decrease in ocular discomfort symptoms. This can be related to the properties of hyaluronic acid with lubricating properties, forms a viscoelastic solution in water, and contributes to create a mechanical protection for cells and to protective effects of trehalose due to its capacity to protect cellular membranes and labile proteins against damage and denaturation [8, 9].

According to the literature and our study it is evident that the use of a tear substitute is of benefit after surgery [13, 18–20]. We observed an improvement in puncture sensations in both groups at two and four weeks, but the greater effectiveness of the trehalose/sodium hyaluronan eye drops is confirmed by the difference between the values observed at 4 weeks in the majority of the testing we carried out. Of note, we observed an improvement in ocular symptoms and OSDI two weeks after surgery, which is quicker than that recorded in a similar study using a carboxymethylcellulose 0.5%/HA 0.1% formulation, where signs and symptoms were improved after four or five weeks [13].

The improvements in dry eye signs and symptoms observed in the study group indicate that the use of trehalose/HA eye drops improves ocular surface health, which is consistent with their properties. HA’s properties of retaining water and hydrating the ocular surface make it a useful means of treating ocular surface disorders [21], whilst trehalose is effective in maintaining the integrity of the phospholipid bilayers, preserving labile cell proteins against desiccation, and protecting cells against oxidative damage [7, 9]. Trehalose also has protective effects on corneal and conjunctival epithelial cells, accelerates corneal healing, reduces the levels of conjunctival inflammatory cytokines, and helps to restore osmotic balance to the ocular surface [22]. In addition, it is already established that trehalose offers significant benefits in the treatment of dry eye compared saline and hydroxy methylcellulose eye drops [8, 23].

The main limitation of this study was the short follow-up period, which makes conclusions on the long-term efficacy of 3% trehalose/ 0.15% sodium hyaluronate eye drops on the signs and symptoms of dry eye difficult. In addition, in the absence of a control group that used either trehalose or hyaluronic acid eye drops individually, our protocol does not allow us to determine if the improvements we observed would have been replicated if patients had received these molecules separately.

Overall, these study results suggest that a 3% trehalose/0.15% sodium hyaluronate gel is effective in reducing DED symptoms after cataract surgery. This study can help to improve post-operative management of patients undergoing cataract surgery and help obtain a better understanding of the role of artificial tears in post-operative care.

## Data Availability

Data of manuscript will not be deposited.
